# Impaired endo-lysosomal membrane integrity accelerates the seeding progression of α-synuclein aggregates

**DOI:** 10.1038/s41598-017-08149-w

**Published:** 2017-08-09

**Authors:** Peizhou Jiang, Ming Gan, Shu-Hui Yen, Pamela J. McLean, Dennis W. Dickson

**Affiliations:** 1 Department of Neuroscience, Mayo Clinic, Jacksonville, FL 32224 USA; 2 Department of Laboratory Medicine and Pathology, Mayo Clinic, Jacksonville, FL 32224 USA

## Abstract

In neurodegenerative diseases, seeding is a process initiated by the internalization of exogenous protein aggregates. Multiple pathways for internalization of aggregates have been proposed, including direct membrane penetration and endocytosis. To decipher the seeding mechanisms of alpha-synuclein (αS) aggregates in human cells, we visualized αS aggregation, endo-lysosome distribution, and endo-lysosome rupture in real-time. Our data suggest that exogenous αS can seed endogenous cytoplasmic αS by either directly penetrating the plasma membrane or via endocytosis-mediated endo-lysosome rupture, leading to formation of endo-lysosome-free or endo-lysosome-associated αS aggregates, respectively. Further, we demonstrate that αS aggregates isolated from postmortem human brains with diffuse Lewy body disease (DLBD) preferentially show endocytosis-mediated seeding associated with endo-lysosome rupture and have significantly reduced seeding activity compared to recombinant αS aggregates. Colocalization of αS pathology with galectin-3 (a marker of endo-lysosomal membrane rupture) in the basal forebrain of DLBD, but not in age-matched controls, suggests endo-lysosome rupture is involved in the formation of αS pathology in humans. Interestingly, cells with endo-lysosomal membrane permeabilization (LMP) are more vulnerable to the seeding effects of αS aggregates. This study suggests that endo-lysosomal impairment in neurons might play an important role in PD progression.

## Introduction

Intracellular deposits of filamentous α-synuclein(αS) aggregates, termed Lewy bodies and Lewy neurites, are a common pathological feature of Parkinson’s disease (PD) and related disorders^1, 2^. Although the pathogenesis of Lewy body disorders remains elusive, cell-to-cell transmission of αS aggregates has been proposed to be responsible for the spread and progression of αS pathology^3–5^. Evidence to support this hypothesis comes from several *in vitro* and in *vivo* studies in which recombinant αS aggregates have been shown to be capable of seeding the polymerization of endogenous intracellular αS by acting as a nuclei for aggregation^6–8^. Seeding activity has also been confirmed in homogenates of mouse and postmortem human brains with αS pathology^7, 9, 10^. The exact mechanisms associated with αS seeding remain to be determined. Seeding is a process initiated by the internalization of exogenous seeds consisting of aggregated proteins. Multiple pathways, including direct membrane penetration and endocytosis, have been proposed from studies of different types of protein aggregates^11–14^; however, direct evidence for the pathways responsible for αS internalization is still lacking. One interesting question is whether membrane rupture of endo-lysosomes is responsible for the seeding activity of αS aggregates since Freeman *et al*. have reported that exogenous αS fibrils can induce lysosome rupture following endocytosis^15^. To date no evaluation of αS seeding activity and pathways involved in the internalization of αS aggregates have been reported. Understanding how αS enters cells is important to understand the progression of αS pathology in human brains. To compare αS aggregates derived from recombinant αS (*in vitro*) with those from postmortem human brains (*in vivo*) with Lewy pathology, we employed cell models capable of visualizing αS aggregation, endo-lysosome distribution, and endo-lysosome rupture in real-time to monitor the seeding process of each preparation. The present study confirms both direct plasma membrane penetration- and endocytosis-mediated αS seeding pathways, and provides further information towards an in-depth understanding of the seeding mechanism of αS aggregates formed *in vitro* and *in vivo*. Most importantly, for the first time we reveal a new role for endo-lysosome impairment in the propagation of αS aggregates in human cells, which may have important implications for the progression of αS pathology in neurons in PD and related diseases, and thus could open the door to innovative therapeutic options.

## Materials and Methods

### Cell culture and maintenance

Three cell models were used in the present study. The first one was H4/V1S-SV2, a previously described H4 neuroglioma cell line that inducibly co-expresses the N-terminal half of Venus YFP tagged αS (V1S) and C-terminal half of Venus YFP tagged αS (SV2) upon TetOff induction^16^. The second model – H4/V1S-SV2/Lamp1-eCFP – is derived from H4/V1S-SV2 and infection with lentivirus carrying Lamp1-eCFP, which was further used to establish a third model – H4/V1S-SV2/Lamp1-eCFP/mCherry-Galectin-3 – via infection with lentivirus carrying mCherry-Galectin-3. All three cell models were maintained in OPTI-MEM (Invitrogen) medium supplemented with 10% fetal bovine serum (Invitrogen), 200 µg/ml hygromycin B, 200 µg/ml G418 and 2 μg⁄ml tetracycline (Tet) and incubated at 37 °C. Cells were seeded in media without Tet at a density of 1 × 10^4^ cells/well in Nunc® Lab-Tek® II chambered coverglasses (Sigma-Aldrich) for live cell imaging, or 2 × 10^4^ cells/well on coverslips in 24-well plates for immunocytochemistry. Experiments were performed after at least 4 days of TetOff induction.

### Lentiviral plasmids and virus preparation

Lentiviral plasmid carrying Lamp1-eCFP [Ex-T8016-Lv154] was purchased from Genecopoeia, and mCherry-Galectin-3, “pLVX-mCherry-Galectin-3” was a gift from Dr. Edward M. Campbell (University of Illinois at Chicago). The preparation of lentivirus was described previously^17^.

### Preparation of *in vitro* formed αS seeds

Recombinant human αS was freshly prepared as previously described^18^. The αS fibrils for seeding were prepared in accordance with two previous reports^19, 20^ with minor modifications. Briefly, purified αS was diluted in aggregation buffer [10 mM phosphate, 2.7 mM KCl, and 137 mM NaCl, pH 7.5] to a final concentration of 300 μM, then incubated at 37 °C with constant shaking in a Thermomixer. After 7 days (for preparing smaller seeds) or 14 days (for preparing larger seeds), fibrils were pelleted by ultracentrifugation (110,000 × g, 20 min), resuspended in the same buffer. The solution was aliquoted and stored at −80 °C. Prior to use, aliquots were completely sonicated for 2 minute using Sonicator 3000 (Misonix) for generation of small seeds. To evaluate the aggregation pattern and morphology, a small volume of sonicated seeds was subjected to both SDS-PAGE/western blotting and electron microscopy (EM).

### Preparation of *in vivo* formed αS seeds

To prepare *in vivo* αS seeds and controls, the amygdala was dissected from frozen brains of 3 patients with diffuse Lewy body disease (DLBD) and 3 age-matched controls. Brains were acquired from the brain bank for neurodegenerative disorders at Mayo Clinic in Jacksonville, FL. The tissue samples were homogenized in sterile PBS with tissue glass Dounce homogenizer. Homogenates were cleared by centrifugation for 5 minutes (3,000 × g, 4 °C), and the resulting supernatant (lysate) was aliquoted and stored at −80 °C. Prior to use, the lysates were thawed and completely sonicated for 2 minutes using Sonicator 3000 (Misonix). For purification by immunoprecipitation (IP), the sonicated lysates were incubated with Dynabeads® Protein G (10003D, Thermo Fisher Scientific) crosslinked to α-synuclein monoclonal antibody, clone 4B12 Covance), followed by washing, elution and neutralization.

### Seeding and quantification of intracellular αS aggregates

For seeding experiments, cell media was replaced with 400 µl Opti-MEM (Invitrogen) containing different forms of αS (soluble, sonicated fibrils, brain lysates and IP products). After 2 days incubation at 37 °C, seeded aggregates in cells was monitored either in real-time or after fixation and immunocytochemistry with confocal microscopy (Zeiss LSM 510, Carl Zeiss MicroImaging). For each group in each experiment, five fields (upper left, upper right, center, lower left and lower right) with at least 90 cells were chosen to count the ratio of cells containing seeded αS inclusions. Two single cells from each field of a total of 10 cells were randomly selected for counting seeds associated with different structures.

### Immunocytochemistry

Cells grown on cover slips were rinsed with PBS, fixed in 4% paraformaldehyde and permeabilized with 0.1 M Tris-buffered saline (TBS; pH 7.6) containing 0.5% Triton X-100 for 5 minutes, then blocked with 3% goat serum in TBS, followed by incubation with primary antibodies in TBS containing 1% goat serum overnight at 4 °C then with secondary antibodies for 1 hour. Immunolabeled cells were stained with nuclear stain DAPI (Invitrogen) for 10 minutes (for H4/V1S-SV2 cells only) and observed with confocal fluorescence microscopy.

### Immunofluorescent staining

Sections of paraffin embedded tissue were subjected to deparaffinization, rehydration, steaming in DAKO target retrieval solution pH 6.1 for 30 minutes, and incubation at room temperature with Protein Block (X0909, DAKO) for 1 h. For immunofluorescent staining, the sections were incubated with primary antibodies to αS (NACP98, 1:500, rabbit polyclonal^21^) and Galectin-3 (1:250, mouse monoclonal, Biolegend) at 4 °C overnight, followed by 1.5 hours of incubation with secondary antibodies (1:500) after washing. Non-specific fluorescence signals were blocked by staining with Sudan Black. Sections were coverslipped with Vectashield mounting media (H-1200, Vector Laboratories, Burlingame, CA) and viewed with confocal microscopy.

### Statistical analysis

Data from at least 3 independent experiments were analyzed by one-way ANOVA with Dunnett’s *post hoc* test for comparison of more than 3 groups or Student’s t test for comparison of two groups. A p-value of < 0.05 was considered statistical significant.

## Results

### Seeding of endo-lysosome-free and endo-lysosome-associated αS inclusions by exogenous αS fibrils

**Figure 1:**
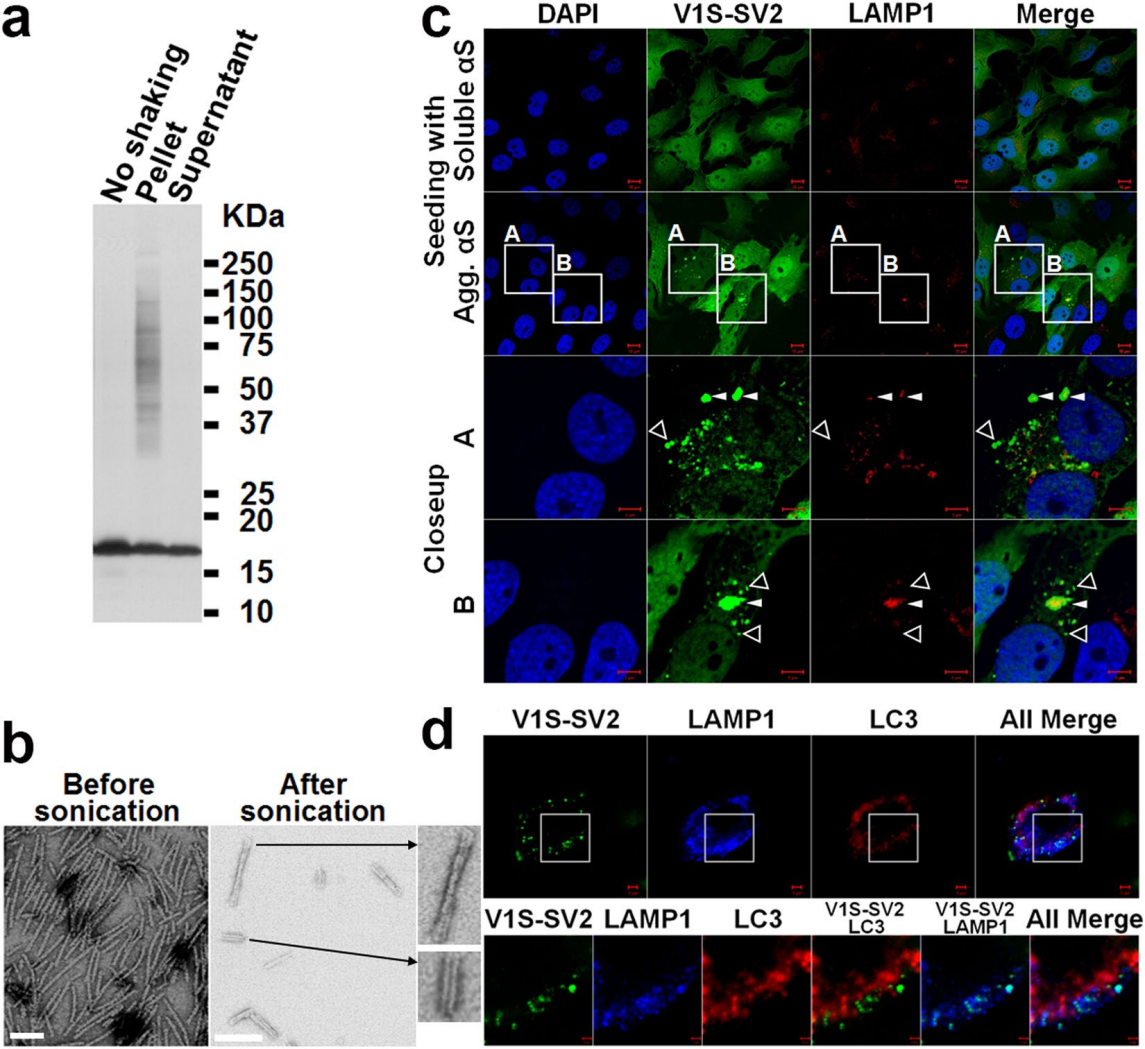
I*n vitro* αS seeds induce endo-lysosome-free and endo-lysosome-associated αS inclusions in H4/V1S-SV2 cells. **(a)** Gel analysis of *in vitro* prepared αS seeds. Recombinant αS solution subjected to 7 days of shaking was ultracentrifuged (110,000 × g, 20 min), and the resultant supernatant and pellet were resolved by SDS-PAGE and western blotting with anti-αS antibody (Synuclein-1, 610787, BD Biosciences). A second aliquot without shaking was included as a control. **(b)** Electron micrographs of αS seeds before and after sonication. Scale bar: 100 nm. Different αS polymorphs are shown in close-ups. **(c)** H4/V1S-SV2 cells treated with sonicated αS seeds for 2 days were subjected to immunocytochemistry with primary antibody against LAMP1 followed by DAPI counterstain to demonstrate the association between endo-lysosomes and seeded αS inclusions. The bottom two rows are magnified images of frames A and B in the second row. Empty triangles and solid arrowheads denote the endo-lysosome-free and endo-lysosome-associated αS inclusions, respectively. Scale bar: 10 µm (top two rows) and 5 µm (bottom two rows). **(d)** The treated cells were further subjected to immunocytochemical staining of LAMP1 (Alexa fluor 405) and LC3 (Alexa fluor 568) to demonstrate the association between autophagosomes, endo-lysosomes, and seeded αS inclusions. Scale bar: 5 µm (top row) and 2 µm (bottom row).

It is thought that αS seeding requires both interaction of internalized exogenous seeds (αS aggregates) and interaction with endogenous, intracellular αS. Although evidence to support αS seeding is still lacking, it has been proposed that exogenous pathogenic seeds might enter recipient cells by directly penetrating the plasma membrane or by endocytosis to initiate seeding^11–14^^,^^22^. Accordingly, we hypothesized that αS inclusions will have a distinct cellular distribution depending on the pathway of internalization of the seeds. For example, we predicted that αS inclusions formed from seeds internalized by endocytosis, a membrane-associated process, would co-localize with endo-lysosomes; whereas those formed from direct plasma penetration (independent of membrane processes) would not. To investigate the different seeding pathways we used an H4 neuroglioma cell-derived cell line – H4/V1S-SV2 – as described previously^16^. This cell line inducibly co-expresses the N-terminal half of Venus YFP tagged to αS (V1S) and C-terminal half of Venus YFP tagged αS (SV2). Upon TetOff induction, αS aggregation can be monitored in real-time because the interaction of V1S with SV2 upon αS-αS interaction can reconstitute fluorescence^23^. The filamentous αS aggregates used for seeding were confirmed by SDS-PAGE/western blot and EM (Fig. 1a and b). H4/V1S-SV2 cells were treated with sonicated αS fibrils for 2 days to induce intracellular αS aggregates as detected by the formation of fluorescent (YFP) inclusions, then subjected to immunocytochemistry with an antibody (sc-20011, mouse, Santa Cruz Biotechnology) against endo-lysosome-associated membrane protein 1 (LAMP1, a marker of late endosome and endo-lysosome^24, 25^) to demonstrate the colocalization of endo-lysosome with seeded αS inclusions. We observed that αS fibrils induced both endo-lysosome-free ad endo-lysosome-associated αS inclusions (Fig. 1c), consistent with our prediction and a previous study reported by Tsujimura, *et al*.^26^. Because the seeded αS inclusions were not immunopositive for LC3 (NB600-1384, Rabbit, Novus Biologicals) or p62 (NBP1-48320, Rabbit, Novus Biologicals) as shown in Fig. 1d and Supplementary Fig. S1, the endo-lysosome-associated αS inclusions are unlikely to be in a process of autophagolysosomal degradation. These results strongly support endo-lysosome-associated αS inclusions are seeded via an endocytosis pathway.

**Figure 2:**
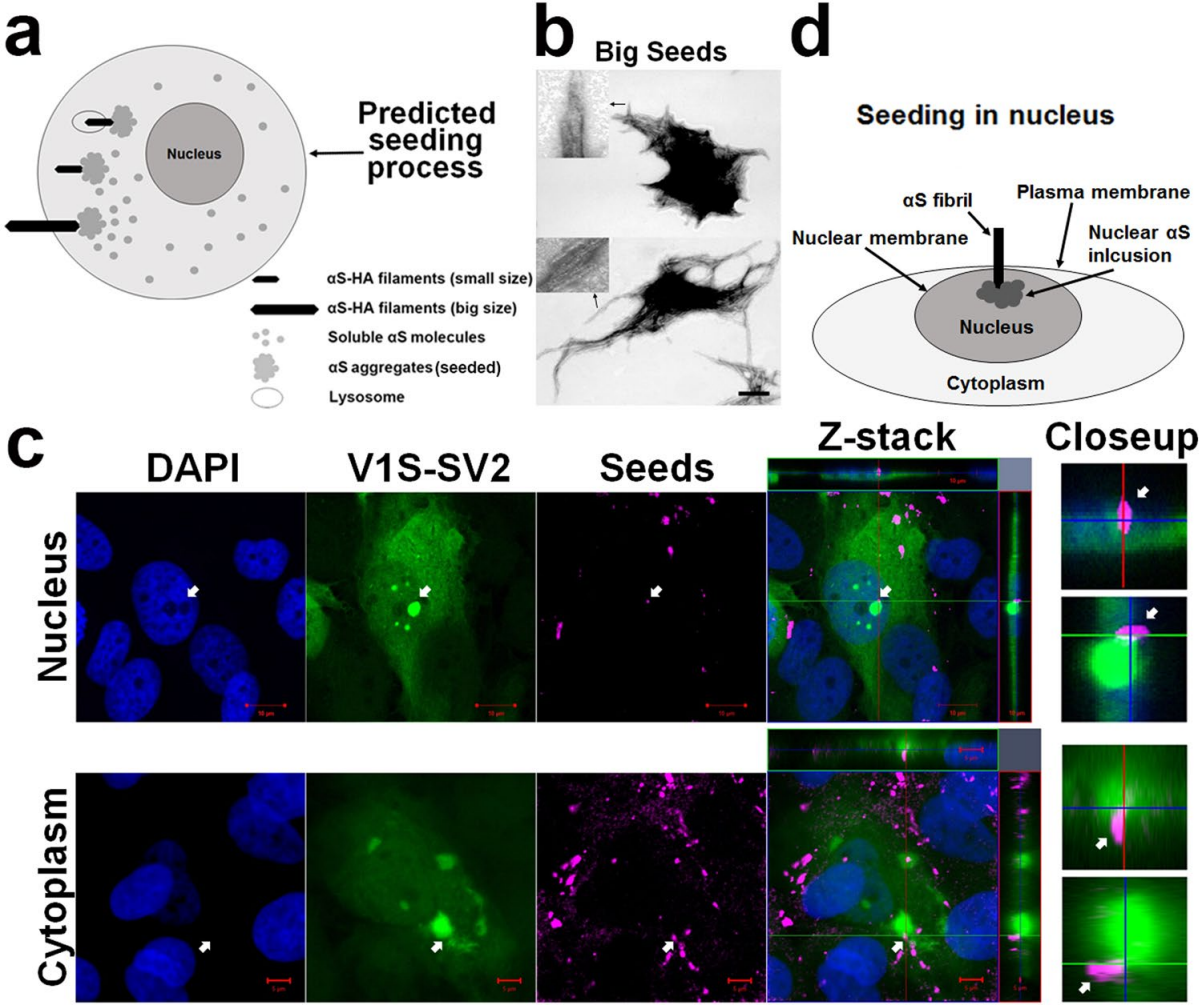
Evidence for a direct plasma membrane penetration seeding pathway. **(a)** Schematic of predicted seeding processes by small and large seeds. **(b)** Electron micrographs of large seeds. Scale bar: 500 nm. Different αS polymorphs are shown in two magnified inserts. **(c)** H4/V1S-SV2 cells were treated with αS-HA seeds for 2 days then subjected to immunocytochemistry with primary antibody against HA tag and Alexa Fluor 647-conjugated secondary antibody. Z-stack of merged pictures confirms the intracellular location of seeded inclusions and partial-internalization of large seeds in recipient cells. Arrows denote the seeded αS inclusions formed in nucleus (top row) and cytoplasm (bottom row). Scale bar: 10 µm.

Compelling evidence that endo-lysosome-free αS inclusions are induced by seeds internalized via a direct plasma membrane penetration pathway is still lacking. To address this issue, we prepared large αS aggregates by briefly sonicating mature fibrils of recombinant αS-HA fusion protein (αS-HA) described previously^18^. Because large fibrils are inefficiently internalized, if a direct plasma membrane penetration pathway exists, we expected to see partially-internalized αS-HA filaments with one end projecting into the cell cytoplasm and associated with seeded intracellular αS inclusions and the other end protruding outside of cells as depicted in (see Fig. 2a **)**. The filamentous morphology of large fibrils with width and length of 2~3 µm was confirmed by EM (Fig. 2b). H4/V1S-SV2 cells were treated with fibrils for 2 days then subjected to immunocytochemistry with an antibody against the HA tag. Immunostaining revealed HA positive filaments with one end partially internalized and co-localized with a cluster of aggregates in the cytoplasm, and the other end protruding into the extracellular space (bottom row in Fig. 2c as depicted in Fig. 2a), supporting the existence of direct plasma membrane penetration. Interestingly, seeded αS inclusions could also be found in the cell nucleus (top row in Fig. 2c). We observed a partially internalized HA-immunopositive αS seed colocalizing with a seeded nuclear αS inclusion. Because it is unlikely that exogenous seeds enter the nuclei of cells via endocytosis, a reasonable explanation is that nuclear αS inclusions are induced by larger αS-HA filaments via direct penetration through both plasma and nuclear membranes to seed aggregation of nuclear αS as depicted in Fig. 2d.

**Figure 3:**
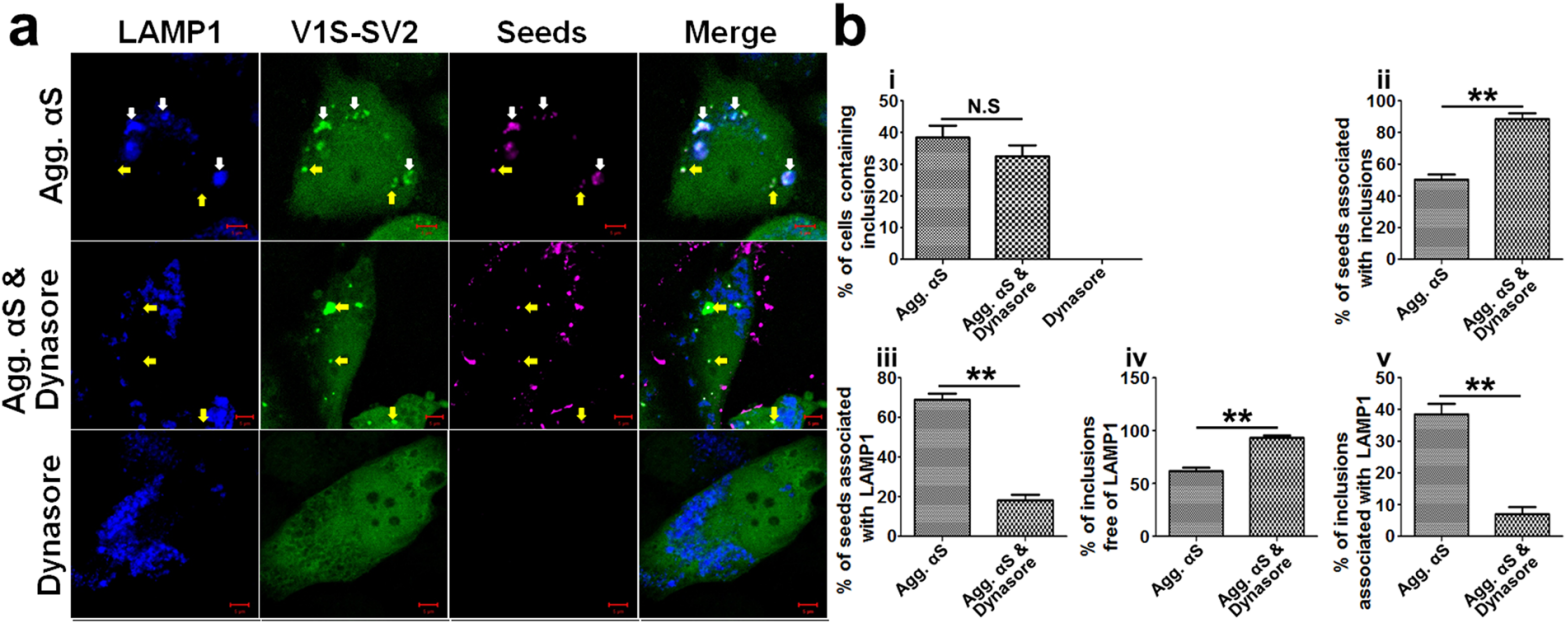
Membrane penetration- and endocytosis-mediated αS seeding contribute to formation of endo-lysosome-free and endo-lysosome-associated αS inclusions. (**a**) H4/V1S-SV2/LAMP1-eCFP cells were respectively treated with DyLight® 650 labeled αS seeds (Agg. αS), Dynasore, and Dynasore & Agg. αS for 2 days followed by imaging under confocal microscopy. The concentration for Agg. αS is 0.5 µg/ ml, and Dynasore 20 µM. Yellow and white arrows denote αS seeds and induced LAMP1-free (yellow), LAMP1-positive (white) αS inclusion, respectively. Scale bar: 5 µm. (**b**) Bar graphs show the comparisons among Agg. αS, Dynasore, and Dynasore & Agg. αS treated cells in the ratio of cells with inclusions to total cells, inclusions to total seeds, LAMP1-positive seeds to total seeds, LAMP1-negative and LAMP1-positive inclusions to total inclusions, respectively. Error bars represent standard error of the mean (N.S = non-significant; **p < 0.01, comparing subsets linked by line, n = 3).

To further support the existence of a membrane penetration pathway, we treated H4/V1S-SV2 cells with endocytosis blocker *Dynasore*, a cell-permeable, reversible non-competitive dynamin inhibitor, before adding αS fibrils. Endocytosis inhibition did not significantly decrease the ratio of cells containing inclusions, but significantly decreased the ratio of endo-lysosome-associated αS seeds and inclusions, with a concomitant increase in endo-lysosome-free αS seeds and inclusions (see Fig. 3). These results indicate that an endocytosis-mediated αS seeding contributes to formation of endo-lysosome-associated αS inclusions and supports endo-lysosome-free αS inclusions being induced by exogenous seeds via a direct membrane penetration pathway.

### αS seeds internalized via direct plasma membrane penetration and endocytosis have distinct seeding activity

**Figure 4:**
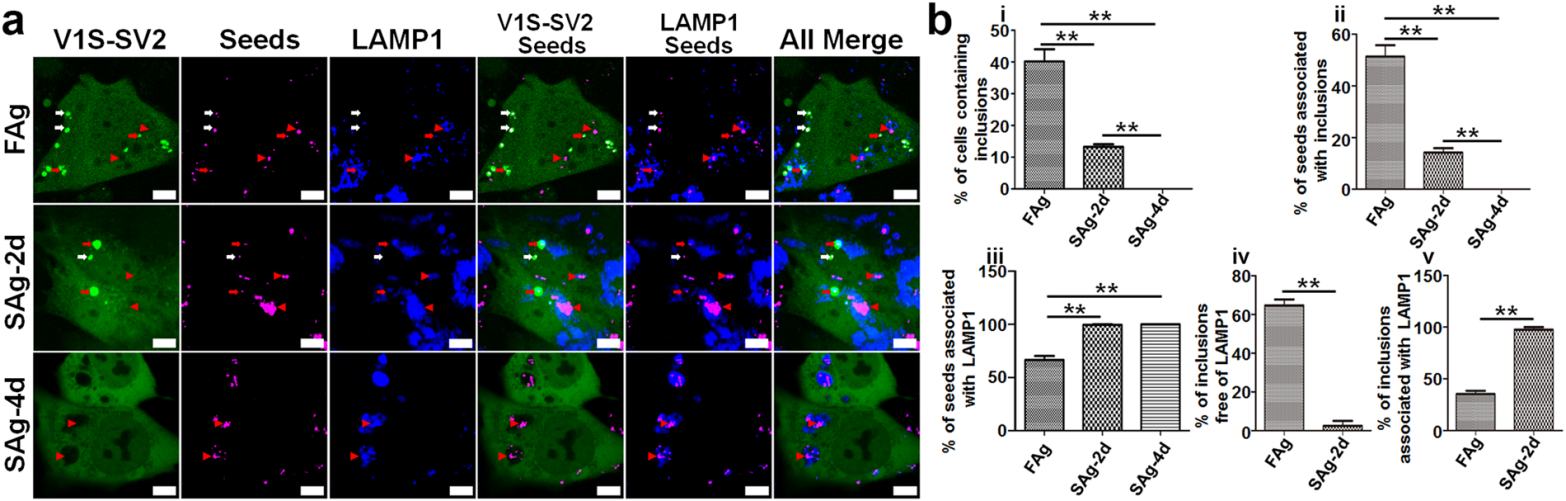
Seeding activity is related to pathway of αS seeds. **(a)** H4/V1S-SV2/LAMP1-eCFP cells were treated with DyLight® 650 labeled FAg, SAg-2d, or FAg-4d for 2 days followed by imaging under confocal microscopy. White and red arrows respectively denote αS seeds and induced endo-lysosome-free (white), endo-lysosome-associated (red) αS inclusions; red arrowheads denote endo-lysosome-associated αS seeds without induction of an αS inclusion. Scale bar: 5 µm. **(b)** i, ii &iii: quantitation of FAg, SAg-2d, and SAg-4d treated cells expressed as the ratio of cells with inclusions to total cells, inclusions to total seeds, and LAMP1-positive seeds to total seeds; iv &v: quantitation of FAg and SAg-2d treated cells expressed as the ratio of LAMP1-negative and LAMP1-positive inclusions to total inclusions. Error bars represent standard error of the mean (*p < 0.05, **p < 0.01, comparing subsets linked by line, n = 3).

In theory, seeding via a direct plasma membrane penetration pathway should be faster and more potent than via endocytosis because the former is independent of membrane processes while the latter must go through multiple steps, including transport to endo-lysosomes and then release into the cytoplasm where it can template endogenous αS. We rationalized that seeds with preferential formation of endo-lysosome-free aggregates via a direct plasma membrane penetration would have higher seeding potency. Since we and others have observed that seeding activity of αS fibrils drastically decreases upon storage at 4°C^20, 27^, we tested whether the observed decreases were related to a particular seeding pathway. Two aliquots of αS fibril stocks were thawed and incubated at 4°C for 2 and 4 days, referred to as SAg-2d and SAg-4d (SAg standing for stored aggregates), to differentially reduce their seeding activities. An aliquot freshly thawed from 80°C was used as positive control, referred to FAg (freshly thawed aggregates). To monitor the association between endo-lysosome and seeded αS inclusions, we established a stable cell line expressing V1S-SV2 and LAMP1-eCFP. FAg, SAg-2d and SAg-4d were labeled with Lightning-Link® Rapid DyLight® 650 (Innova Biosciences), then equal amounts of each sample (0.5 µg/ml) were used to treat H4/V1S-SV2/LAMP1-eCFP cells for 2 days followed by imaging under confocal microscopy. Exogenous αS seeds, intracellular seeded inclusions, and endo-lysosomes are represented by fluorescence of Dylight 650, bright YFP, and eCFP, respectively. Compared to FAg, SAg-2d induced significantly less αS inclusions, and SAg-4d failed to induce any inclusions (Fig. 4a and graph i and ii in Fig. 4b), suggesting that storage at 4°C can reduce seeding activity in a time-dependent manner. Moreover, we found that seeds in cells treated with SAg-4d co-localized with endo-lysosomes (graph iii in Fig. 4b), and the ratio of endo-lysosome-free αS inclusions to total inclusions in cells treated with FAg was significantly higher than that with SAg-2d (Fig. 4a and graph iv and v in Fig. 4b). These data suggest a positive correlation between seeding activity and the capability of αS seeds to induce endo-lysosome-free αS aggregates. Therefore, it is likely that seeding ability is related to the preferential seeding pathway of the seeds. These data demonstrate how external factors, such as inappropriate storage, can drastically affect the preferential internalization pathway of the seeds, leading to a partial reduction or even complete loss of the seeding activity.

### Endo-lysosome rupture precedes formation of αS inclusions in endocytosis-mediated seeding

Regardless of which internalization pathway is involved, interaction between exogenous seeds and cytoplasmic αS is required for seeding to take place. Such an interaction is easy to understand with direct plasma membrane penetration of the fibril. For endocytosis-mediated seeding, however, it is unclear how exogenous seeds interact with cytoplasmic αS. The main function of endocytosis in mammalian cells is to internalize molecules from the plasma membrane and sort them to endo-lysosomes for degradation. For successful seeding by exogenous seeds, an interaction needs to occur with cytoplasmic αS before being degraded in endo-lysosomes. Such a scenario is not possible unless the endo-lysosome membranes rupture and seeds escape into the cytoplasm. Freeman *et al*. have reported that exogenous αS fibrils can induce the accumulation of Galectin-3, a marker of endo-lysosome rupture, following endocytosis^15, 28^. To determine if, endo-lysosome rupture is associated with interaction of seeds and cytoplasmic αS, we monitored the accumulation of Galectin-3 in the endo-lysosome membrane of fibril-treated cells^29–31^.

**Figure 5:**
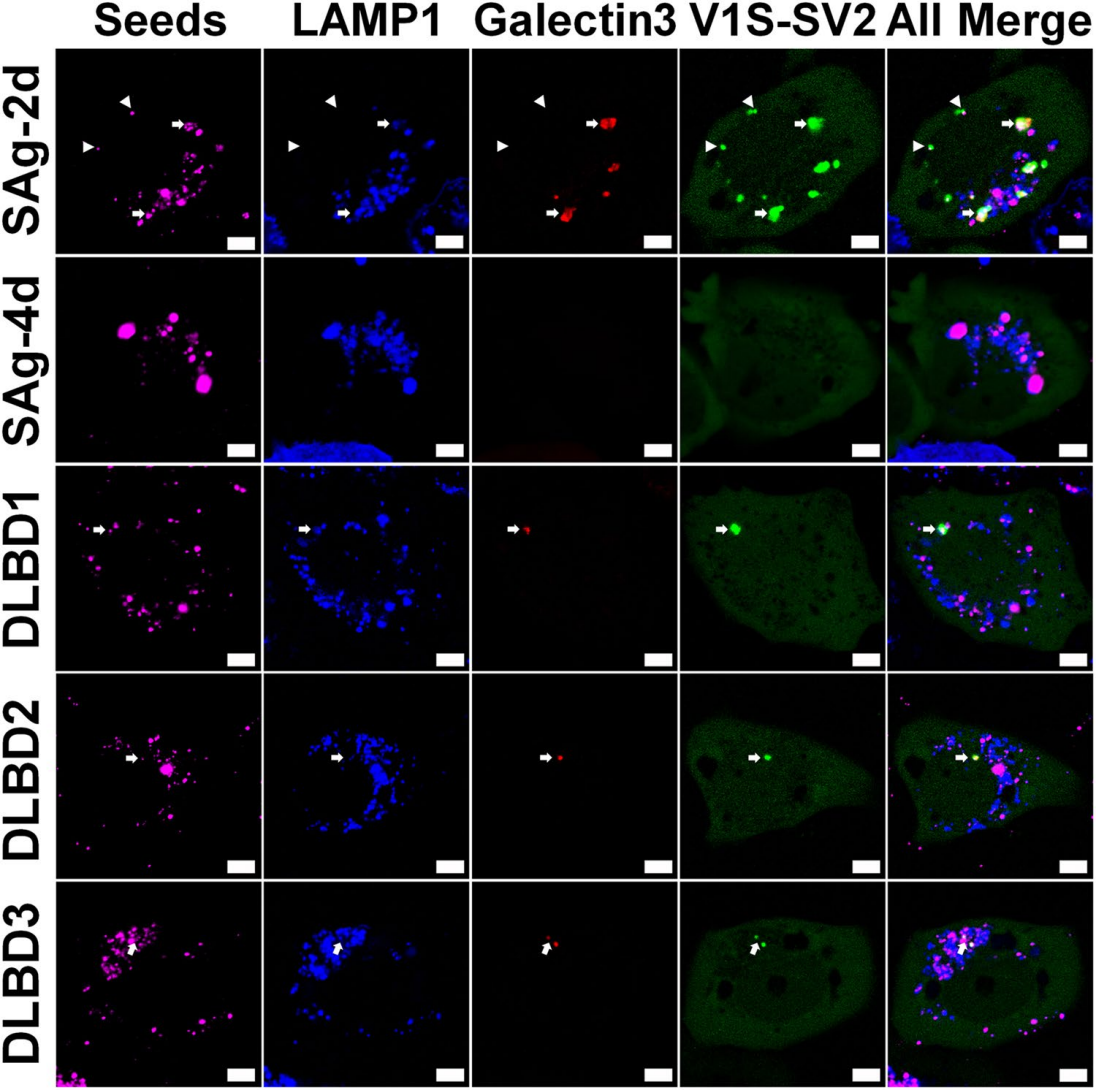
Endo-lysosome rupture is involved in endocytosis-mediated seeding. H4/V1S-SV2/ LAMP1-eCFP/mCherry-Galectin-3 cells were treated with DyLight® 650 labeled SAg-2d, SAg-4d, or immunoprecipitation-isolated αS from brain lysates of 3 different DLBD cases (DLBD1, DLBD2 and DLBD3) for 2 days followed by imaging under confocal microscopy. Arrows denote αS seeds and their induced αS inclusions associated with endo-lysosomes and Galectin-3 accumulation. Arrowheads denote αS seeds and their induced αS inclusions that are not associated with endo-lysosome and Galectin-3. Scale bar: 5 µm. Table 1. Quantification of the seeds associated with different structures in different groups.

To investigate membrane rupture, we introduced mCherry fused to N-terminus of Galectin-3 (mCherry-Galectin-3) into H4/V1S-SV2/LAMP1-eCFP cells via lentivirus infection. The stable cell model is referred to as H4/V1S-SV2/LAMP1-eCFP/mCherry-Galectin-3. Cells were treated with SAg-2d or SAg-4d labeled with Lightning-Link® Rapid DyLight® 650 (Innova Biosciences) for 2 days, then observed under confocal microscopy. Interestingly, intracellular SAg-2d seeds are either free or associated with LAMP1 (Fig. 5, row 1 & 2 in Table 1); and all LAMP1-free seeds are associated with seeded inclusions, but not Galectin-3 (arrowheads in Fig. 5, row 3 & 4 in Table 1). LAMP1-associated seeds appear as two types: one that is not associated with seeded inclusions (Fig. 5, row 5 in Table 1) and one that is associated with both seeded inclusions and Galectin-3 (arrows in Fig. 5, row 6 in Table 1), indicative of the involvement of endo-lysosome membrane rupture in endocytosis-mediated seeding. In contrast, all intracellular SAg-4d seeds were associated with LAMP1, but no seeded inclusions could be observed (Fig. 5, row 2 & 6 in Table 1), which is consistent with decreased SAg-4d seeding activity.

**Table 1:** Quantification of the seeds associated with different structures in different groups in Fig. 4.

		SAg-2d	SAg-4d	DLBD1	DLBD2	DLBD3
1	The ratio of seeds (LAMP1−) to total seeds	3.1 ± 2.8	0	0	0	0
2	The ratio of seeds (LAMP1+) to total seeds	96.9 ± 2.8	100	100	100	100
3	The ratio of seeds (LAMP1−/inclusion+) to seeds (LAMP1−)	100	N/A	N/A	N/A	N/A
4	The ratio of seeds (LAMP1−/inclusion+/Galectin−3+) to total seeds	0	0	0	0	0
5	The ratio of seeds (LAMP1+/inclusion−) to seeds (LAMP1+)	87.5 ± 2.7	100	96.6 ± 3	97.2 ± 2.4	96 ± 3.5
6	The ratio of seeds (LAMP1+/inclusion+) to seeds (LAMP1+)	12.5 ± 2.7	0	3.4 ± 3	2.8 ± 2.4	4 ± 3.5
7	The ratio of seeds (LAMP1+/inclusion+/Galectin-3+) to seeds (LAMP1+/inclusion+)	100	N/A	100	100	100

Note: All values are percentile.

**Figure 6:**
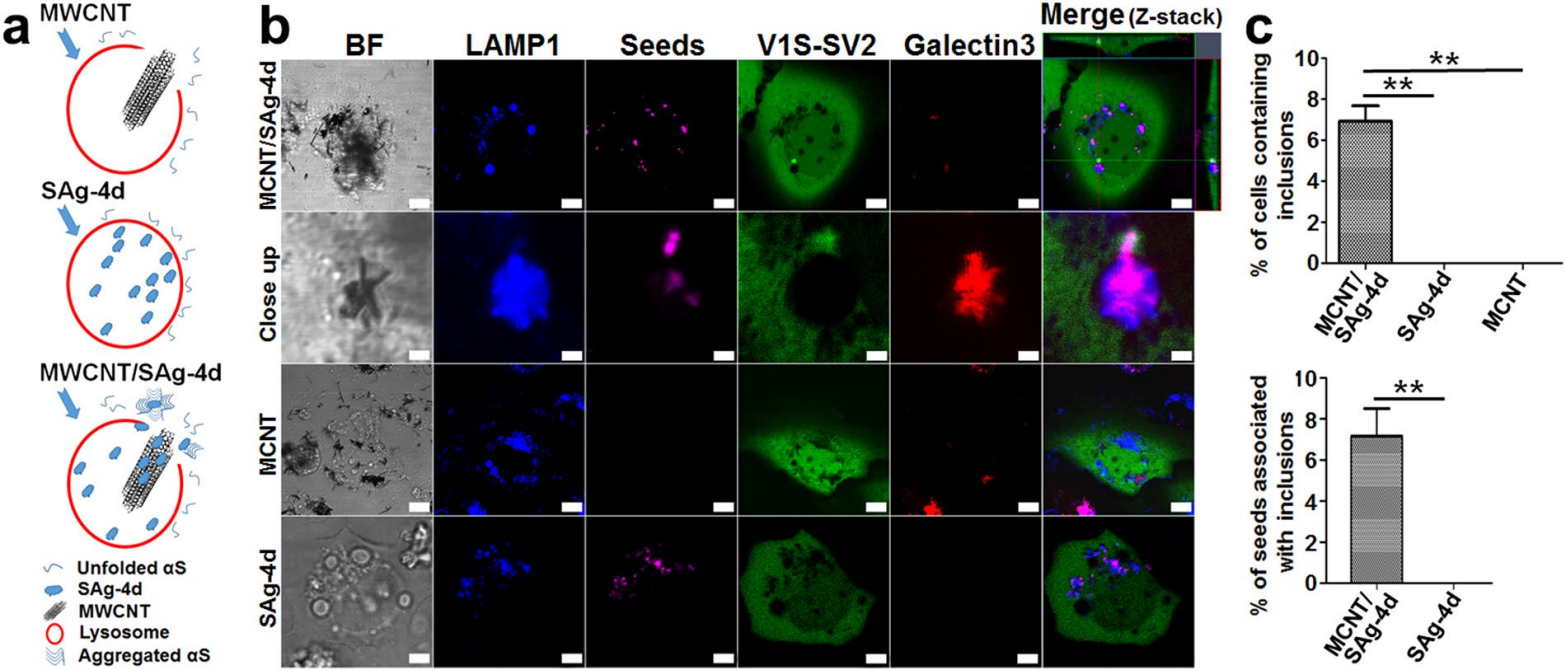
MWCNT partially recovers seeding activity of SAg-4d via induction of endo-lysosome rupture. **(a)** Schema of predicted effects of MWCNT on facilitating seeding of SAg-4d. **(b)** H4/V1S-SV2/LAMP1-eCFP/mCherry-Galectin-3 cells were treated with the mixture of MWCNT and DyLight® 650 labeled SAg-4d for 2 days followed by imaging under confocal microscopy. For controls, cells treated with MWCNT or DyLight® 650 labeled SAg-4d alone were used. The framed areas in pictures of the first row (MCNT/SAg-4d) are enlarged and shown in the second row (Close up). Z-stack of images in MCNT/SAg-4d group confirmed the intracellular location of seeded αS inclusion. Scale bar: 1 µm for the second row and 5 µm for all the rest. **(c)** The comparison among MCNT/SAg-4d, SAg-4d and MCNT treated cells in the ratio of cells with inclusions to total cells is shown as the top bar-graph, and that between MCNT/SAg-4d and SAg-4d treated cells in the ratio of inclusions to total seeds shown as the bottom bar-graph. Error bars represent standard error of the mean (*p < 0.05, **p < 0.01, comparing subsets linked by line, n = 3).

Next we mixed SAg-4d with multiwall carbon nanotubes (MWCNTs, 100 nm diameter, Sigma) and treated H4/V1S-SV2/LAMP1-eCFP/mCherry-Galectin3 cells. Carbon nanotubes are allotropes of carbon with a cylindrical nanostructure that can enter cells through endocytosis and readily penetrate endo-lysosome membranes with their tubular structure and extremely high aspect-ratio^32^. We predicted that MWCNTs, when co-localized with SAg-4d in the same endo-lysosome, would facilitate SAg-4d dissolution of endo-lysosome membrane (see schema in Fig. 6a). As predicted, cells treated with a mixture of MWCNT/SAg-4d formed seeded αS inclusions compared to cells treated with SAg-4d or MWCNTs alone, indicating that the seeding activity of SAg-4d partially recovered in the presence of MWCNT (Fig. 6b and c). It is worth noting that, the endo-lysosome membrane encapsulating MWCNT/SAg-4d showed Galectin-3 where the end of single nanotubes were apposed to the membrane, indicative of membrane penetration. Interestingly, exogenous αS seeds and associated αS inclusions could be observed immediately adjacent to endo-lysosome membranes with accumulated Galectin-3, indicating seeding by it escaping from ruptured endo-lysosome facilitated by the MWCNT (Fig. 6b). These results strongly support the hypothesis that MWCNT-induced endo-lysosome membrane rupture directly contributed to endocytosis-mediated seeding.

**Figure 7:**
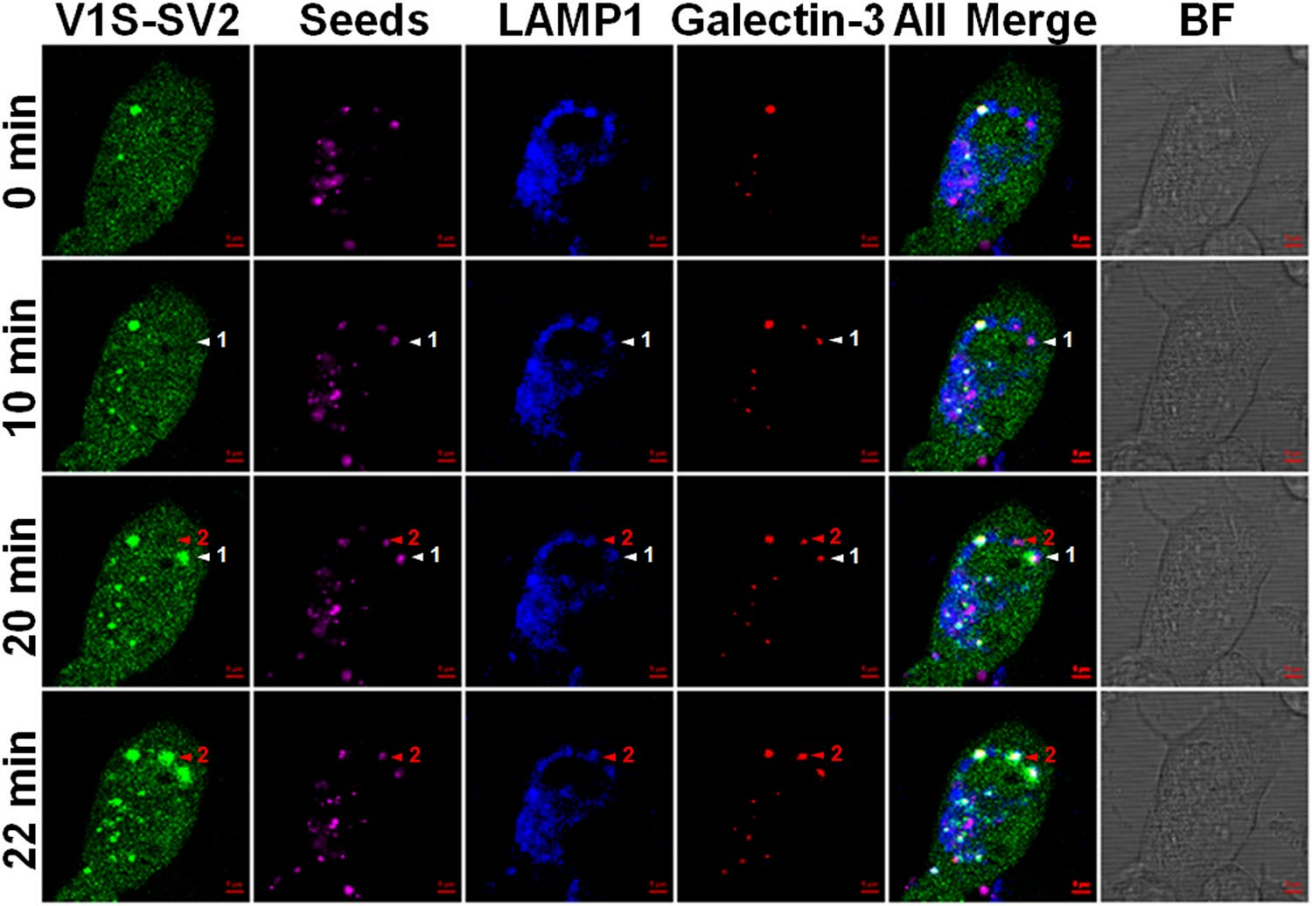
Endo-lysosome rupture precedes formation of αS inclusions in endocytosis-mediated seeding. H4/V1S-SV2/LAMP1-eCFP/mCherry-Galectin-3 cells were incubated with DyLight® 650 labeled SAg-2d for 2 days then a single cell forming αS inclusions was chosen for time-lapse imaging under confocal microscopy. White and red arrowheads respectively denote the sequential events during the formation of two different αS inclusions (No. 1 and No. 2). Scale bar: 1 µm.

To determine if membrane rupture precedes the formation of seeded αS inclusions, H4/V1S-SV2/LAMP1-eCFP/mCherry-Galectin-3 cells were incubated with labeled SAg-2d then subjected to time-lapse imaging by confocal microscopy (Zeiss LSM 510, Carl Zeiss MicroImaging, Pleasanton, CA) at 37 °C to monitor the sequential events of seeding. Imaging revealed that accumulation of Galectin-3 appears first, followed by formation of seeded inclusions. Moreover, the time interval between accumulation of Galectin-3 and appearance of seeded inclusions was as little as 2 minutes (Fig. 7), indicating that seeding is a rapid process. These data support an important role for endo-lysosome rupture in endocytosis-mediated seeding by releasing exogenous seeds into the cytoplasm to template intracellular endogenous αS.

### Pathological αS aggregates isolated from brains with Lewy body pathology preferentially show endocytosis-mediated seeding

**Figure 8:**
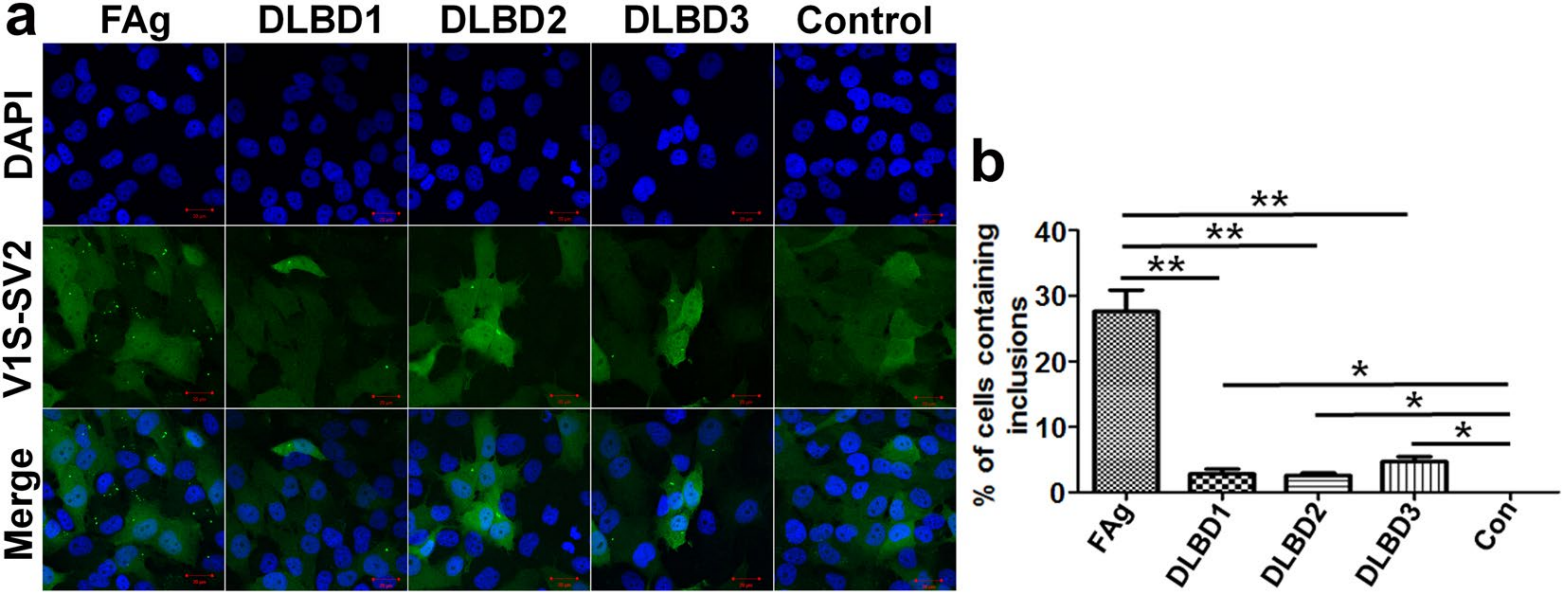
The comparison of seeding activity of αS seeds from human brain and *in vitro* recombinant αS. **(a)** H4/V1S-SV2 cells were treated with 0.1 µg αS from IP/αS products isolated from amygdala of 3 patients with DLBD (DLBD1, DLBD2 and DLBD3) and 1 age-matched control (Control), and an aliquot of FAg for 2 days followed by imaging under confocal microscopy. Scale bar: 20 µm. **(b)** The bar graph shows the comparison of the ratio of cells with inclusions to total cells among all groups.

To determine if the seeding activity and pathway of αS aggregates formed from recombinant protein *in vitro* and isolated form human brain are comparable, αS was immunoprecipitated from the amygdala of 3 patients with DLBD (referred to as DLBD1, DLBD2 and DLBD3) and one age-matched control. The concentration of αS in each sample was determined using ELISA (KHB0061, Thermo Fisher Scientific). The same amount (0.1 µg) of αS or control sample was added into H4/V1S-SV2 cells for 2 days; then, the number of cells containing seeded αS inclusions from each group was counted and compared. Interestingly, cells bearing seeded inclusions in FAg groups were significantly higher than all other groups (Fig. 8). There was no significant difference in seeding activity between all three DLBD patients, although DLBD3 showed highest seeding activity (Fig. 8b).

To investigate the seeding pathway, H4/V1S-SV2/LAMP1-eCFP/mCherry-Galectin3 cells were treated with DyLight® 650 labeled DLBD samples for 2 days then imaged with confocal microscopy. All internalized seeds were found to be associated with endo-lysosomes. Moreover, seeded αS inclusions were not only associated with endo-lysosomes, but also with mcherry-Galectin-3 (see row 2 and 6 in Table 1), indicative of the involvement of endocytosis and endo-lysosome rupture in the seeding. Compared to αS seeds from recombinant protein, αS from human brain has significantly less seeding activity and appears to preferentially use an endocytosis-mediated pathway.

### Endo-lysosome rupture is involved in the progression of αS pathology in Lewy body disease

**Figure 9:**
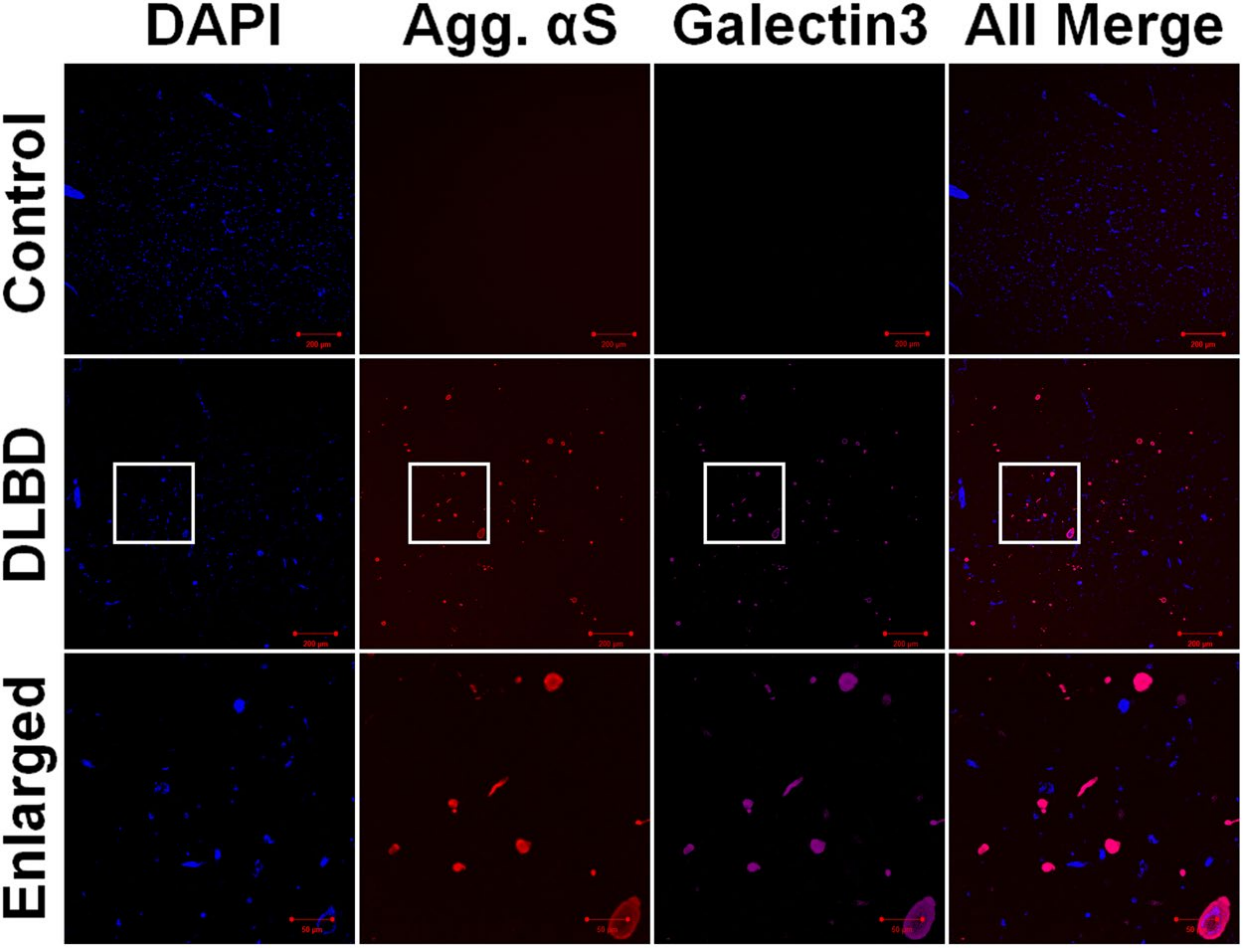
Galectin-3 co-localizes with αS pathology in Lewy body disease. Human specimens containing basal nucleus of Meynert from four patients with DLBD and four age matched controls were evaluated with double-labeling immunofluorescence and antibodies to αS and Galectin-3. The framed areas in the second row (DLBD) are enlarged and shown in the third row (Enlarged). Scale bar: 200 µm.

To determine if endocytosis-mediated seeding and endo-lysosome rupture are involved in the progression of αS pathology *in vivo*, we investigated Galectin-3 co-localization with αS pathology in Lewy body disease. Brain sections containing basal nucleus of Meynert from 4 patients with DLBD and 4 age-matched controls were subjected to dual immunofluorescence with antibodies to Galectin-3 and αS. Co-localization of Galectin-3 and αS pathology was observed in all 4 DLBD cases, but not in normal controls (Fig. 9). These results support the hypothesis that endo-lysosome rupture is involved in the progression of αS pathology in Lewy body disease.

### The loss of endo-lysosome membrane integrity renders cells more vulnerable to αS aggregate seeding

**Figure 10:**
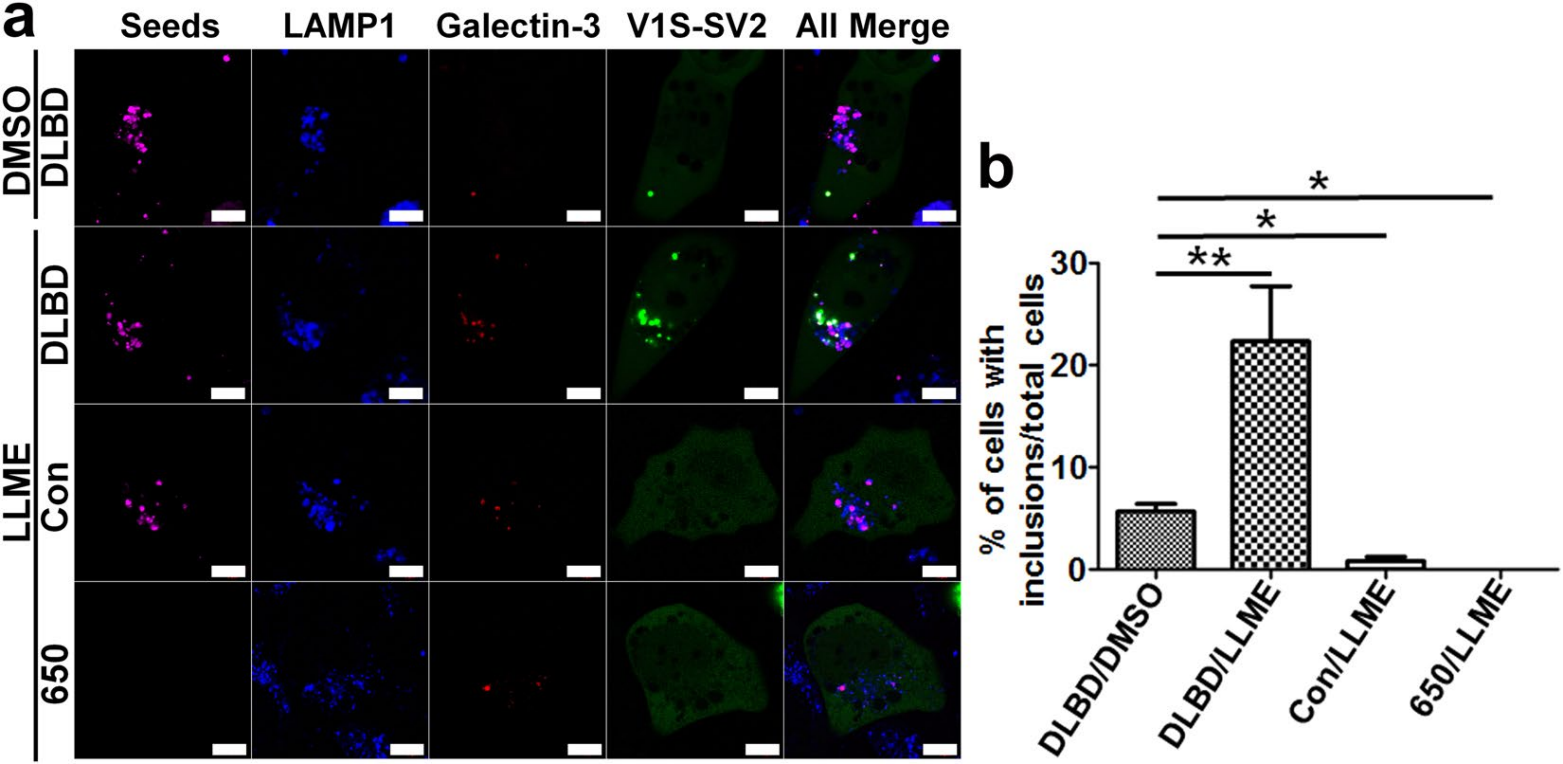
The loss of endo-lysosome membrane integrity renders cells more susceptible to αS aggregates seeding. **(a)** H4/V1S-SV2/LAMP1-eCFP/mCherry-Galectin-3 cells were treated with DyLight® 650 (or labeled αS immunoprecipitates from lysates of DLBD3 (DLBD) and an age-matched control (Con)) for 2 days, then exposed to L-Leucyl-L-Leucine methyl ester (LLME) for 3 hours followed by imaging under confocal fluorescence microscopy. A counterpart of the DLBD sample was treated with only the vehicle (DMSO) as a non-LLME control. **(b)** A bar graph shows the comparison of the ratio of cells with inclusions to total cells among all groups. **(**Table 2 **)** Quantification of the ratio of the seeds associated with different structures in different groups in **(a)**.

Because brain αS aggregates prefer endocytosis-mediated seeding pathway (Fig. 5) and disruption of endo-lysosome membranes can facilitate seeding (Fig. 6), and since lysosome membrane permeabilization (LMP) of neurons is considered important in pathogenesis of PD^33, 34^, we hypothesized that cells with compromised endo-lysosome membrane integrity might be more susceptible to αS aggregation after internalizing αS seeds via endocytosis. To test this hypothesis, we treated H4/V1S-SV2/LAMP1-eCFP/mCherry-Galectin-3 cells with labeled αS seeds from DLBD3 for 2 days, then exposed cells to a lysosomotropic detergent – L-Leucyl-L-Leucine methyl ester (LLME) – for 3 hours. LLME can be internalized by cells through endocytosis, then converted into (LeuLeu)n-OMe (n > 3) by dipeptidyl peptidase I (Cathepsin C) in lysosomes, leading to lysosome rupture^35^. In parallel, cells were treated with DLBD3 alone, LLME plus DLBD3, LLME plus DyLight® 650 labeled lysates from age-matched control (Con), or LLME plus Lightning-Link® Rapid DyLight® 650. Using confocal fluorescence microscopy, we observed a significant increase in αS inclusions in cells treated with DLBD3 upon exposure to LLME (Fig. 10). Moreover, all seeded inclusions were associated with both endo-lysosomes and accumulated Galectin-3 (Table 2). By contrast, no intracellular aggregates were detected in LLME/Con and LLME/650 treated groups (Fig. 10a and b). These results suggest that loss of endo-lysosome membrane integrity favors the release of endocytic αS seeds into the cytoplasm and renders cells more susceptible to seeding of αS aggregates.

**Table 2:** Quantification of the ratio of the seeds associated with different structures in different groups in Fig. 9

		DLBD/ DMSO	DLBD/ LLME	Con/ LLME	650/ LLME
1	The ratio of seeds (LAMP1−) to total seeds	0	0	0	N/A
2	The ratio of seeds (LAMP1+) to total seeds	100	100	100	N/A
3	The ratio of seeds (LAMP1+/inclusion−) to seeds (LAMP1+)	95.5 ± 2	81.4 ± 4.9	100	N/A
4	The ratio of seeds (LAMP1+/inclusion+) to seeds (LAMP1+)	4.5 ± 2	18.6 ± 4.9*	0	N/A
5	The ratio of seeds (LAMP1+/inclusion+/Galectin-3+) to seeds (LAMP1+/inclusion+)	100	100	N/A	N/A

Note: All values are percentile. *P < 0.01 versus DLBD/DMSO.

## Discussion

In the present study, we demonstrate that exogenous αS aggregates can enter recipient cells by direct plasma membrane penetration or by endocytosis to initiate seeding. Based on the observation that seeded αS aggregates are either associated with endo-lysosome or not, we suggest there are distinct seeding pathways. Endo-lysosome-free αS aggregates are induced by a direct plasma membrane penetration pathway, while endo-lysosome-associated αS aggregates are induced through an endocytosis-mediated pathway. In order to understand the mechanisms underlying endocytosis-mediated seeding, we monitored association of Galectin-3 as a marker of membrane rupture and demonstrated that seeds can template cytoplasmic αS to form aggregates by rupturing endo-lysosome membranes during trafficking into endo-lysosomes (Figs 5–7). Therefore, for both seeding pathways, membrane rupture is an indispensable step occurring early in the process, when seeds contact plasma membranes or after seeds are transported into endo-lysosomes.

It is intriguing why some seeds prefer to directly penetrate the plasma membrane while others choose to rupture endo-lysosomes via endocytosis. It is well-known that the composition of different membranes varies throughout cells, leading to their various biological functions. Plasma membranes are enriched in sphingolipids and sterols (e.g., cholesterol) which are packed at a higher density than glycerolipids andresist mechanical stress^36^. The high concentration of sphingolipids and cholesterol make the plasma membrane an impermeable barrier between the cytoplasm and cell exterior^37^. Early endosomes are similar to plasma membrane, but on maturation to late endosomes and lysosomes, there is a significant change in the composition of membranes, especially in the reduction of cholesterol^36^. Since the proportion of cholesterol in bilayers is positively correlated to the thickness and impermeability of membranes^38^, the reduction of cholesterol in the membranes of late endosomes and lysosomes makes them more permeable than plasma membrane and early endosomes. Therefore, it is reasonable to deduce that the preferred seeding pathway is dependent on membrane penetration ability of the seeds. If robust enough, seeds can induce endo-lysosome-free αS aggregates through direct plasma membrane penetration pathway; if partially reduced and only strong enough to penetrate endo-lysosome membrane, the seeds enter cells through endocytosis first, then induce endo-lysosome-associated αS aggregates via membrane penetration. If the structure of seeds is too weak to penetrate membrane structures, they are incapable of inducing αS aggregates even after uptake into endo-lysosomes through endocytosis. Accordingly, the loss of seeding activity after incubation at 4°C as demonstrated in the present study is associated with reduced potency for membrane penetration. It is worth noting that Bousset *et al*. reported that a certain type of αS fibrils can dissociate into monomers upon storage at 4°C^27^. One may argue that such dissociation might be potentially account for reduced seeding activity of the αS aggregates in our study. However, we believe this is not the case. Evidence is that the protocol for αS fibril preparation in our study is quite different and the product may not be the same; we also observed a considerable amount of twisted filaments in the preparations (see Fig. 1b and Fig. 2b) resembling irreversible αS ribbons, which are stable structures that would not be predicted to dissociate upon storage at 4°C; Finally, the loss of seeding activity of “SAg-4d” is unlikely due to the reversible dissociation to monomeric αS, otherwise, it would be impossible to recover seeding activity using multiwall carbon nanotubes and the lysosomotropic detergent LLME. Therefore, dissociation of fibrils is unlikely to be the key reason underlying loss of seeding activity in our study. Although the exact reason remains unclear, it might be comparable to an empirical phenomenon of a razor blade becoming blunt over time due to inappropriate storage even without being used.

Although we confirmed that plasma membrane penetration and endocytosis are two major pathways responsible for exogenous αS seeding, it remains to be determined how the plasma membrane interacts with αS seeds in response to each seeding pathway. In this regard, previous studies have shown that several proteins on the cell surface can interact with αS fibrils. For examples, heparan sulfate proteoglycans^14^ and lymphocyte-activation gene 3 can facilitate the internalization and transmission of αS fibrils via endocytosis^39^, and neuronal a3-Na + /K + -ATPase can be sequestered by αS aggregates leading to impairment of Na + gradient^40^. Additional studies will be needed to determine whether or not these proteins have any effect on membrane penetration or endo-lysosome rupture of αS seeds.

It is worth noting that in contrast to many other studies, no internalization-facilitating reagents, such as wheat germ agglutinin or transfection reagents were used in this study^8, 26^. Interestingly, we found that the αS aggregates from 3 DLBD cases had significantly reduced seeding activity compared to freshly thawed *in vitro fibrils*. Similarly, in a previous study Woerman *et al*. used αS aggregates purified from 9 brains with Lewy body pathology (6 PD with and without dementia and 3 DLBD), and they failed to observe any intracellular αS aggregates in cell cultures^41^. Therefore, the seeding activity of αS aggregates from Lewy body brain samples may be significantly less than that of aggregates prepared *in vitro* from recombinant αS. The possibility remains, however, that there might be other αS-associated components in brain homogenates that interfere with seeding activity and decrease their ability to penetrate plamsa or endo-lysosomal membranes. In this regard, compared to αS fibrils formed *in vitro*, αS pathology is formed over a long term process during which many complicated molecular events may be occurring. For example, interaction with other proteins such as tissue transglutaminase, rab3a, and rabphilin ^42^ ^43^, post-translational modifications such as ubiquitination^44, 45^ and phosphorylation^46, 47^, are all changes that may potentially alter the interaction between αS aggregates and plasma membrane leading to the impairment of preferential seeding pathway and seeding activity.

We further demonstrated that the pathological αS from brains with Lewy body disease preferentially seeds αS aggregates in human cell cultures by the endocytic pathway and endo-lysosome rupture. Importantly, we found that co-localization of Galectin-3 and αS pathology in the basal forebrain of DLBD, but not in normal, age-matched controls. These data strongly suggest that endo-lysosome rupture is likely involved in the seeding and transmission of αS pathology in the brains of PD and related disorders.

Finally, the use of LLME to induce LMP highlighted the fact that cells with LMP are more vulnerable to seeding of exogenous αS aggregates. Although in recent years lysosomal impairment, including LMP, has been increasingly recognized as a key event in PD pathogenesis^48–51^, the predominant view regarding the underlying mechanism is related to its contribution to the accumulation or aggregation of endogenous αS due to deficient degradation. Our present study is the first report to demonstrate that endo-lysosomes with compromised integrity are liable to be ruptured by endocytic seeds leading to release of αS into the cytoplasm for seeding. Confirmation of these data in primary neurons will be important to confirm relevance to PD. Nevertheless, this study raises the possibility that LMP in neurons not only causes accumulation of endogenous αS due to impairment of endo-lysosomal degradation, but may also facilitate the progression of αS pathology due to the favorable effects on seeding of exogenous αS aggregates in the brain in PD and related disorders.

Overall, this study provides novel insights into the mechanisms underlying seeding of αS aggregates in human cells, and expands the understanding of the role of lysosomal impairment in the propagation of αS aggregates. If our current study is reproducible in primary neurons, preservation of endo-lysosomal membrane integrity or induction of endo-lysosomal biogenesis could be a potential upstream target to decelerate or halt the progression of αS pathology in PD and related disorders.

## Electronic supplementary material

supplemental material
