# Supplementary material for: Impaired endo-lysosomal membrane integrity accelerates the seeding progression of α-synuclein aggregates
Source: Sci Rep. 2017 Aug 9;7:7690. doi: 10.1038/s41598-017-08149-w (PMC5550496; doi:10.1038/s41598-017-08149-w)
Supplement: Supplementary file 1 — supplemental material [file 41598_2017_8149_MOESM1_ESM.pdf]

Impaired endo-lysosomal membrane integrity accelerates the seeding progression of α-synuclein aggregates

Peizhou Jiang[1], Ming Gan[1, 2], Shu-Hui Yen[1], Pamela J. McLean[1],

and Dennis W. Dickson[1]

1. Department of Neuroscience, Mayo Clinic, Jacksonville, FL 32224
2. Department of Laboratory Medicine and Pathology, Mayo Clinic, Jacksonville, FL 32224

Supplementary information

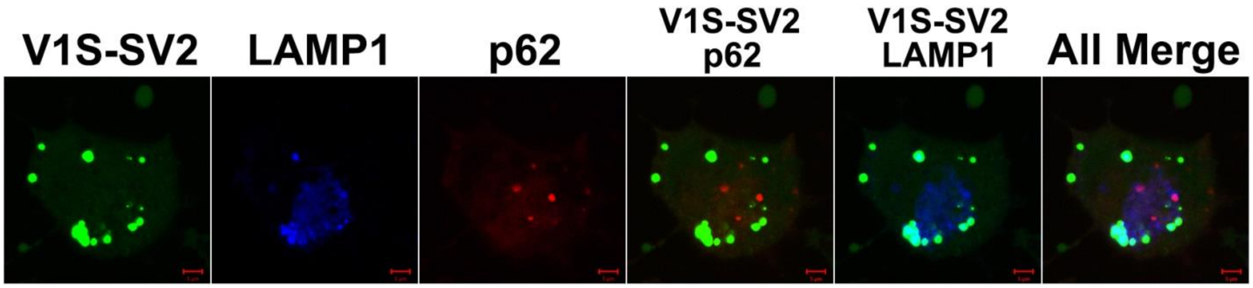

Supplementary Figure S1. H4/V1S-SV2 cells treated with sonicated αS seeds for 2 days were subjected to immunocytochemistry staining with LAMP1 (Alexa fluor 405) and p62 (Alexa fluor 568) to demonstrate the association between p62, endo-lysosomes and seeded αS inclusions. Scale bar: 5 µm.
